# Cardiac helical CT involving a low-radiation-dose protocol with a 100-kVp setting: Usefulness of hybrid iterative reconstruction and display preset optimization

**DOI:** 10.1097/MD.0000000000005459

**Published:** 2016-11-18

**Authors:** Yuji Iyama, Takeshi Nakaura, Koichi Yokoyama, Masafumi Kidoh, Yasuyuki Yamashita

**Affiliations:** a Diagnostic Radiology, Kumamoto chuo hospital; b Department of Diagnostic Radiology, Graduate School of Medical, Kumamoto University; c Diagnostic Radiology, Amakusa Medical Center, Amakusa, Kumamoto, Japan.

**Keywords:** cardiac CT, display preset optimization, hybrid iterative reconstruction, low-voltage tube technique

## Abstract

To compare the radiation dose and image quality of retrospective electrocardiogram (ECG)-gated cardiac computed tomography (CT) between a 100-kVp protocol, hybrid iterative reconstruction (HIR), and display preset optimization and the 120-kVp protocol.

We prospectively enrolled 100 patients with tachycardia or atrial fibrillation scanned retrospective ECG-gated cardiac CT. We randomly assigned 50 patients to the 120-kVp protocol and 50 patients to the 100-kVp protocol. We compared effective doses (EDs) between the two protocols. The 120-kVp images were post-processed using filtered back projection (FBP). The 100-kVp images were post-processed using FBP (100-kVp protocol) and HIR (i-100-kVp protocol). We compared attenuation of the ascending aorta, signal-to-noise ratio (SNR), and image noise between the 120-kVp, 100-kVp, and i-100-kVp protocols. We performed qualitative image analysis for the 120-kVp and i-100-kVp protocols.

ED of the 100-kVp protocol (4.4 ± 0.4 mSv) was 76% lower than that of the 120-kVp protocol (18.4 ± 0.6 mSv). Attenuations of the 100-kVp (549.1 ± 73.8 HU) and i-100-kVp (550.5 ± 73.7 HU) protocols were higher than that of the120-kVp protocol (437.3 ± 55.7 HU). Image noise of the 100-kVp (53.6 ± 18.5 HU) and i-100-kVp (30.9 ± 8.6 HU) protocols were higher than that of the120-kVp protocol (23.8 ± 5.7 HU). There was no significant difference in SNR and the result of qualitative image analysis between the 120-kVp and i-100-kVp protocols.

The 100-kVp protocol with HIR reduced the 76% radiation dose while preserving the image quality compared with the conventional 120-kVp protocol on retrospective ECG-gated cardiac CT.

## 1 Introduction

The prospective electrocardiogram (ECG)-triggered image acquisition technique is widely used for cardiac computed tomography (CT) angiography (CTA) because of the progression of multi-detector CT.^[1–3]^ On the other hand, the retrospective ECG-gated protocol might be more suitable for patients with arrhythmia or tachycardia compared with prospective ECG-gated protocol because the retrospective ECG-gated protocol can choose the optimized cardiac phase to evaluate the coronary artery accurately.^[4–6]^ However, the radiation dose of the retrospective ECG-gated protocol was higher compared with that of the prospective ECG-gated protocol.^[7,8]^ Although the radiation dose reduction with the retrospective ECG-gated protocol might be important, there have been only a few recent studies on the subject.^[4,9]^

The hybrid iterative reconstruction (HIR) is known to reduce image noise, and low-voltage tube techniques are also known to reduce the radiation dose. The HIR technique, an alternative to the filtered back projection (FBP) reconstruction method, has the advantage of decreasing image noise.^[10,11]^ However, combined the HIR technique and low radiation dose protocol can reduce only 60% while maintaining image quality compared with conventional radiation dose protocol with FBP reconstruction.^[12]^ On the other hand, the low-voltage tube protocol has the advantage of increasing contrast enhancement and reducing the radiation dose,^[13,14]^ however, it increase image noise and arterial CT attenuation on picture archiving and communication system (PACS) viewer system at the same display preset compared with the standard-voltage tube protocol.^[12,15]^

In general, the window setting for CT scanning affect image quality.^[16]^ A large window width can decrease the visibility of image noise and influence image contrast. Indeed, a previous report suggested that display preset optimization (large window width) with a 100-kVp protocol can yield almost same image quality as that with the 120-kVp protocol with standard display preset while reducing the radiation dose 48%.^[17]^

Numerous studies have shown that the combined low-voltage tube and HIR techniques can reduce the radiation dose without decreasing the signal-to-noise ratio (SNR) compared with standard-voltage tube and FBP techniques.^[18,19]^ However, this technique has a disadvantage of yielding over-smoothed image appearance and changing the CT attenuation compared with a conventional protocol.^[20,21]^

To our knowledge, there have been no previous studies of the evaluation of image appearance or visual contrast with combined 100-kVp protocol and the HIR technique with optimized display presets. We hypothesized that combined 100-kVp protocol and the HIR technique with optimized display presets would drastically decrease the radiation dose without changing the image quality compared with 120-kVp CT scan with FBP reconstruction with display preset.

The purpose of this study was to compare the radiation dose and image quality of retrospective ECG-gated cardiac CT between combined 100-kVp protocol with HIR and display preset optimization and the 120-kVp protocol with FBP.

## 2 Materials and methods

Our institutional review board approved this prospective study. Prior informed consent regarding participation in the study was obtained from all patients.

### 2.1 Patients

Between December 2012 and July 2013, a total of 220 patients suspected of having coronary disease and scanned coronary CTA were considered for participation in this prospective study. We measured their serum creatinine level within 3 months prior to the coronary CTA and determined the estimated glomerular filtration rate (eGFR) using the modified diet in renal disease formula of the Japanese Society of Nephrology.^[22,23]^ We also recorded the patient‘s age, body weight (BW), and sex.

The inclusion criteria was follows; elevated heart rate (>70 beats/min) after use of pharmacological rate control and atrial fibrillation. At 5 minutes before CT, each patient received 0.4 to 0.8 mg of nitroglycerin sublingually to dilate the coronary arteries. If the baseline heart rate was 65 bpm or more, metoprolol (5–15 mg) was injected intravenously. Our exclusion criteria were as follows: renal failure (eGFR of <45 mL/min/1.73 m^2^), history of allergic reactions to iodinated contrast media, heart rate <70 beats/min after use of a pharmacological rate control agent, and proven or suspected pregnancy. One hundred twenty patients were excluded in this study. Consequently, we enrolled 100 patients in this study (63 men, 37 women; ages 35–88 years, mean 62.0 years; BW 33–97 kg, mean 60.3 kg).

### 2.2 CT scanning and contrast infusion protocols

All patients were scanned using a 128-row multi-detector CT instrument (Brilliance iCT; Philips Healthcare, Cleveland, Ohio). We randomly assigned 50 patients to our conventional protocol. They were scanned at 120 kVp using a 0.27-s gantry rotation time, 0.14 helical pitch (beam pitch), and 629-mA tube current, the use of the ECG-dependent tube current modulation technique and retrospective ECG gating. We assigned another 50 patients to the low-radiation protocol involving 100-kVp, with 0.27-s gantry rotation, 0.14 helical pitch (beam pitch), and 268-mA tube current, the use of the ECG-dependent tube current modulation technique and retrospective ECG gating. The detailed scan parameters for each protocol are shown in Table 1.

**Table 1:**
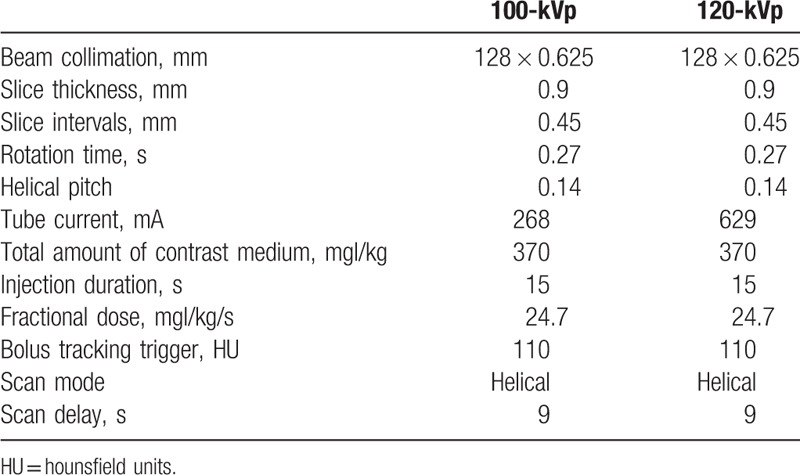
Scan parameters for each protocol.

In all patients, iopamidol 370 mg/mL (Nihon Schering, Osaka, Japan) was delivered over a fixed period of 15 seconds via a 20-gauge catheter inserted into an antecubital vein using a power injector (DUAL SHOT GX; Nemoto-Kyorindo, Tokyo, Japan). The amount of contrast material was adjusted to the body weight of each patient (1 mL/kg). We determined the scan start time using a computer-assisted bolus tracking program (Bolus Pro Ultra; Philips Medical Systems, Cleveland, Ohio)^[24]^ with a trigger threshold of 110 Hounsfield units (HU) in the aortic arch. Real-time (120 and 100 kVp at 15 mAs) serial monitoring studies began 10 seconds after the start of the con**t**rast injection. Scanning started 9 seconds after triggering.

### 2.3 CT image reconstruction

Image reconstruction was performed in a 21.5-cm display field of view. The 120- and 100-kVp images were reconstructed using an FBP algorithm with a Xres standard filter for coronary CT (XCB filter). In addition, a third image set was generated by a 100-kVp protocol reconstructed using HIR (iDose^4^, Philips Healthcare) (i-100-kVp protocol). We selected a hybrid-iterative level of 60% for cardiac imaging, as recommended by the vendor.

### 2.4 Display setting optimization

We set the optimized display setting of the i-100-kVp protocol (window level 260, window width 1040), and the standard display setting of the 120-kVp protocol (window level 200, window width 800). We defined the i-100-kVp protocol with optimized display preset as O-100-kVp protocol. Our optimal window setting was based on that in a previous report, which noted that the 100-kVp protocol for cardiac CT with a display preset of about 1.3 times window level and window width yields almost the same image contrast as that with the 120-kVp protocol.^[17]^

### 2.5 Radiation dose exposure using each protocol

To estimate the CT radiation dose, we recorded the CT volume dose index (CTDI_vol_) and the doselength product (DLP). The effective dose (ED) for coronary CTA was derived from the product of the DLP and a conversion coefficient for the chest in accordance with the European Commission guidelines on quality criteria in CT (*k* = 0.014 mSv mGy^−1^ cm^−1^).^[4]^

### 2.6 Quantitative image analysis

The following measurements were performed on axial images by a radiologist who had 10 years of experience with cardiac CT. First, the CT attenuations of the ascending aorta were measured in a circular region of interest (ROI) placed in the ascending aorta at the level of origin of the left main trunk (ROI_aorta_). An attempt was made to select an ROI of 400 mm^2^ in the ascending aortanot so small that it would be affected by pixel variability and not so large that it would include the vessel wall or perivascular fat. Second, we recorded the image noise, determined as the standard deviation (SD) of the attenuation value of ascending aorta. Third, we recorded the signal to noise ratio (SNR), determined as SNR = ROI_Ao_/noise. We compared these quantitative parameters between the 120-, 100-, and i-100-kVp protocols.

We also adjusted the display presets of the 120-kVp and i-100-kVp protocols and saved the images in bitmap format in the PACS viewer (Synapse: Fujifilm) to perform another quantitative analysis, including the effect of window optimization. The shade of each pixel (0–255) of these bitmap images reflected the brightness of each pixel in the PACS viewer as soft copy at the given display preset. We transferred all CT images to a personal computer for measurement of the shade and image noise of the two protocols. The reason why we used personal computer to evaluate the effect of window optimization was that CT number and the SD of CT numbers on PACS viewer did not reflect the effect of display preset.

The same radiologist measured the shade of the ROI_aorta_ using the same procedure as that used for the original CT images. We also measured the shade of muscle and fat. The shade of muscle (ROI_muscle_) was measured at the interventricular septum in an area that was as large as possible, avoiding the aortic lumen. We measured the ROI of 25 mm^2^ drawn in a homogeneous region of the subcutaneous fat of the anterior chest wall (ROI_fat_). In addition, we measured the image noise, determined as the standard deviation of the shade of the ROI_muscle_. All measurements involving bitmap images were performed using free ImageJ software (ImageJ, version 1.4.5; http://rsbweb.nih.gov/ij/).

### 2.7 Qualitative image analysis

To evaluate the image quality between the 120 kVp with standard display preset protocol and the O-100-kVp protocol, we performed qualitative image analysis of axial images using a PACS viewer.

Two board-certified radiologists with 8 and 5 years of experience, respectively, with cardiac CT, and who were blinded to the reconstruction algorithms, clinical information, and patient radiation dose, independently graded image contrast, image noise, over-smoothed image appearance, and overall image quality. Readers cannot allow change the window setting of two protocols. They independently graded image contrast and overall image quality using a 4-point scale (1 = unacceptable, 2 = acceptable, 3 = good, 4 = excellent). Image noise and over-smoothed image appearance were graded as: 1 = image noise or over-smoothed image appearance that was unacceptable; 2 = image noise or over-smoothed image appearance that interfered with the diagnosis; 3 = image noise or over-smoothed image appearance that did not interfere with the diagnosis; 4 = no significant image noise and over-smoothed image appearance. Inter-observer disagreement was resolved by consensus.

### 2.8 Statistical analysis

All numerical values are reported as means ± SD. All data were tested for normality using Shapiro-Wilk tests and for equality of variances using Levene tests. CTDI_vol_, DLP, ED, the attenuation values, image noise, and SNR were normal distribution and equal variances, therefore, we used the two-tailed Student *t* test to compare CTDI_vol_, DLP, and ED. We used Dunnett method for multiple comparisons of the attenuation values, image noise, and SNR of the 120-, 100-, and i-100-kVp protocols. Values obtained at 120 kVp were used as the control. We used the two-tailed Student *t* test for comparisons of image noise, ROI_aorta_, ROI_muscle_, and ROI_fat_ in the bitmap image between the 120-kVp and O-100-kVp protocols. The visual scores were non-normal distribution or unequal variance, therefore, the visual scores assigned to the 120-kVp and O-100-kVp images were compared using the Mann–Whitney *U* test. Differences with *P* < 0.05 were considered to be statistically significant. The scale for the kappa coefficients for inter-observer agreement was as follows: <0.20 = poor, 0.21–0.40 = fair, 0.41–0.60 = moderate, 0.61–0.80 = substantial, 0.81–1.00 = near-perfect. Statistical analyses were performed using the free statistical R software (R, version 2.6.1: The R Project for Statistical Computing; http://www.r-project.org/).

## 3 Results

### 3.1 Patient characteristics

There were no significant differences between the 100- and 120-kVp groups with respect to age, sex, and body weight (*P* > 0.05) (Table 2).

**Table 2:**
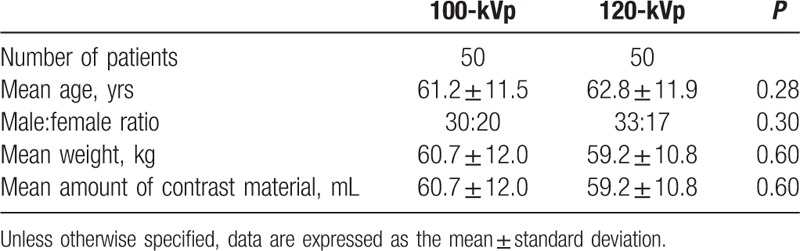
Patient characteristics and contrast agent dose.

### 3.2 Radiation dose

EDs with the 100-kVp protocol were approximately 76% lower than those with the 120-kVp protocol (4.4 ± 0.4 mSv vs. 18.4 ± 0.6 mSv, *P* < 0.01) (Table 3).

**Table 3:**
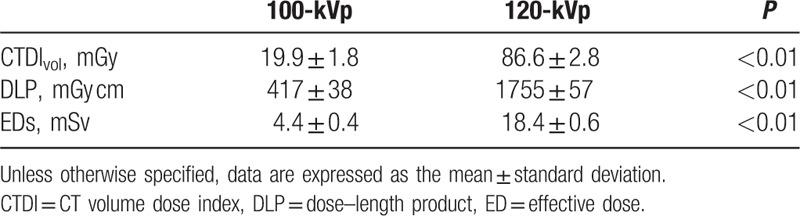
Radiation dose.

### 3.3 Quantitative image analysis

The results of our quantitative image analysis are shown in Table 4. The mean attenuation of the ascending aorta with the 100- and i-100-kVp protocols were significantly higher than that with the 120-kVp protocol (100-kVp vs. 120-kVp protocol: 549.1 ± 73.8 vs. 437.3 ± 55.7 HU [*P* < 0.01]; i-100-kVp vs. 120-kVp protocol: 550.5 ± 73.7 vs. 437.3 ± 55.7 HU [*P* < 0.01]). The mean image noise with the 100-kVp protocol was about 125% higher than that with the 120-kVp protocol: 53.6 ± 18.5 versus 23.8 ± 5.7 HU (*P* < 0.01). The HIR technique decreased the image noise by approximately 42% (100-kVp vs. i-100-kVp protocol: 53.6 ± 18.5 vs. 30.9 ± 8.6 HU [*P* < 0.01]). There was a significant difference in mean image noise between the i-100-kVp and 120-kVp protocols: 23.8 ± 5.7 versus 30.9 ± 8.6 HU (*P* < 0.01). The SNR with the 100-kVp protocol was significantly lower than that with the 120-kVp protocol: 11.1 ± 3.2 versus 19.3 ± 5.1 (*P* < 0.01). There was no significant difference in SNR between the i-100-kVp and 120-kVp protocols: 19.3 ± 5.1 versus 18.9 ± 4.9 (*P* = 0.85).

**Table 4:**
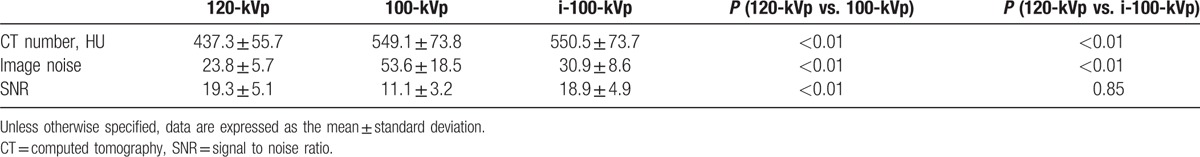
Quantitative image analysis.

The results of the quantitative image analysis of the bitmap images with the 120- and O-100-kVp protocols are shown in Table 5. There was no significant difference in image noise of the bitmap images between the O-100-kVp and the 120-kVp protocols: 4.2 ± 2.3 versus 4.4 ± 3.6 (*P* = 0.70). There were no significant differences in the shade of ROI_aorta_, ROI_muscle_, and ROI_fat_ between the 120-kVp and O-100-kVp protocols: ROI_aorta_: 192.3 ± 22.6 versus 194.9 ± 19.1 (*P* = 0.70); ROI_muscle_: 64.8 ± 7.4 versus 66.6 ± 9.3 (*P* = 0.28); ROI_fat_: 34.1 ± 7.0 versus 36.3 ± 6.5 (*P* = 0.10).

**Table 5:**
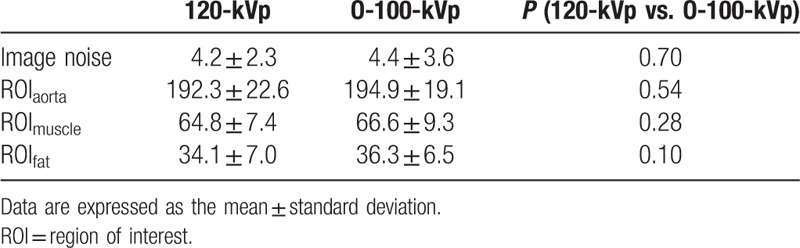
Image noise and shade of bit map images.

### 3.4 Qualitative image analysis

**Figure 1:**
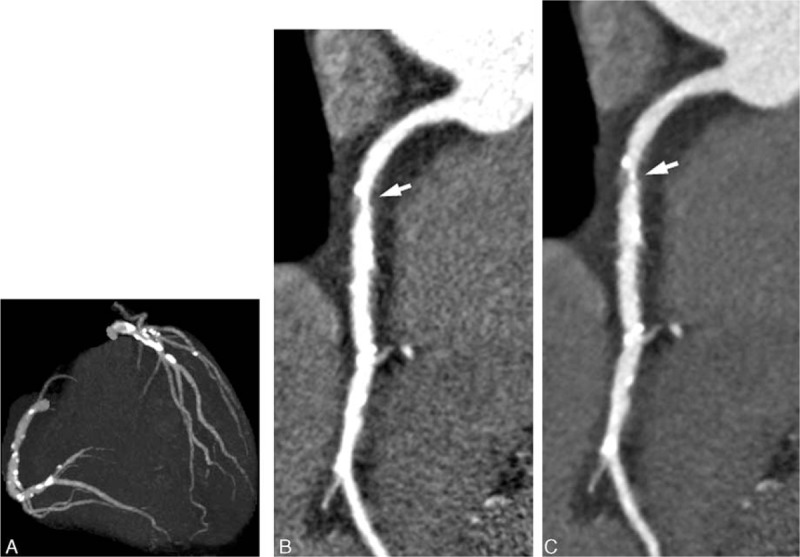
A 73-year-old man with chest pain was suspected of having coronary disease. The patient‘s weight was 56.0 kg at the time of the examination. The i-100-kVp protocol provided remarkable coronary arterial enhancement. The HIR technique decreased the image noise that is seen with the 100-kVp protocol in the maximum intensity projection (MIP) image of coronary CTA (A). Right coronary artery (RCA) stenosis (small arrow) was unclear because of the high image noise and high attenuation of the coronary artery in curved multi-planar reconstructed (MPR) image using the 100-kVp protocol with standard preset (B). By contrast, RCA stenosis (small arrow) in the curved MPR image using the O-100-kVp protocol, was clear. There is lower image noise and appropriate attenuation of RCA in C compared with that in B. CTA = computed tomography angiography, HIR = hybrid iterative reconstruction.

The results of the qualitative image analysis are shown in Table 6. On visual evaluation, there were no statistically significant differences between the 120-kVp and O-100-kVp protocols in regard to image contrast (3.4 ± 0.8 vs. 3.4 ± 0.8, *P* = 0.74); image noise (3.7 ± 0.5 vs. 3.6 ± 0.7, *P* = 0.38); over-smoothed appearance (3.7 ± 0.5 vs. 3.5 ± 0.7, *P* = 0.21); or overall image quality (3.7 ± 0.5 vs. 3.6 ± 0.7, *P* = 0.6). There was substantial to near-perfect inter-observer agreement regarding image contrast, image noise, over-smoothed image appearance, and overall image quality (kappa = 0.79, 0.82, 0.69, and 0.78, respectively). Representative cases are shown in Fig. 1.

**Table 6:**
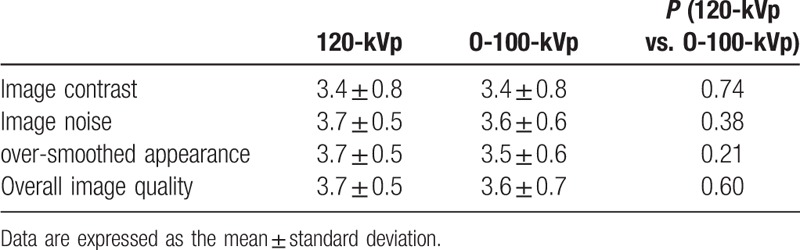
Results of qualitative image analysis regarding the 120-kVp and O-100-kVp protocols (consensus).

## 4 Discussion

Our study suggested that 100-kVp with HIR protocol can reduce 76% radiation dose significantly compared with 120-kVp protocol (4.4 ± 0.4 vs. 18.4 ± 0.6 mSv, *P* < 0.01). However, the image noise and CT attenuation of ascending aorta of 100-kVp with HIR protocol were significantly higher compared with that of 120-kVp protocol. On the other hand, 100-kVp with HIR protocol with optimized display preset can yield same image appearance on bitmap image as 120-kVp with FBP protocol with standard display preset.

There was no significant difference in SNR between 100-kVp with HIR protocol and 120-kVp with FBP protocol. We determined as SNR = ROI_Ao_/image noise. The image noise of 100-kVp with HIR protocol was 29.8% ([30.9–23.8 HU]/23.8 HU) higher compared with 120-kVp with FBP protocol by lowering the tube voltage and tube current. In addition, CT number was also increased by about 26% ([550.5–437.3 HU]/437.3 HU) as a result of the photoelectric effect associated with the 100-kVp scan. Consequently, the SNR for the combined HIR and 100-kVp technique was nearly identical to that with the 120-kVp protocol (ROI_Ao_/image noise = 1.26/1.298).

100-kVp with HIR protocol can reduce 76% radiation dose compared with 120-kVp protocol as expected. We set up a 100-kVp with 268 mAs tube current as a 100-kVp with HIR protocol and 120-kVp protocol with 629 mAs tube current as a 120-kVp protocol. The radiation dose is proportional to the square of the tube voltage.^[25]^ Therefore, with the same tube current, the 100-kVp protocol for cardiac CT can reduce about 41% radiation dose compared with the 120-kVp protocol (1– [100/120]^2^). Additionally, the radiation dose is directly proportional to the tube current. Therefore, 268 mAs tube current protocol with a fixed tube voltage can reduce 57% radiation dose compared with the 629 mAs tube current protocol. We expected that the combined 100 kVp with 268 mAs tube current and HIR technique would reduce the radiation dose by about 75% [1 − (1 − 0.41)  × (1 – 0.57)] compared with the 120-kVp with 629 mAs tube current technique. The estimated radiation dose reduction rate of the combined 100 kVp with 268 mAs tube current compared with the 120-kVp with 629 mAs tube current technique is close to the measured radiation dose reduction rate (75% vs. 76%). In addition, the radiation dose reduction rate of the combined our 100 kVp protocol with HIR with optimized display preset was significantly higher than 100 kVp protocol with optimized display in previous report (76% vs. 48%).^[17]^ The introduction of HIR for low radiation dose protocol can yield significantly radiation dose reduction compared with low radiation dose protocol in previous report.^[17]^

In qualitative image analysis, 100-kVp with HIR can preserve the classic image appearance. Previous studies reported that the classic image appearance defined as the “CT image appearance as known by and familiar to radiologists,” and loss of the classic image appearance may influence the reader's judgement.^[26,27]^ Previous report also suggests that HIR technique cause the over-smoothed appearance and loss of the classic appearance.^[28]^ However, it was not a serious problem when moderate iterative reconstruction was applied.^[21,27]^ The reason why 100-kVp with HIR can preserve the classic image appearance might be that we selected the iDose^4^ level of 60%.

There was no significant difference in the visual contrast of bitmap image between the combined 100-kVp protocol, HIR, and optimal display preset and the 120-kVp protocol with FBP and standard display preset. However, there was a significant difference in the CT number and image noise between the 100-kVp protocol with HIR and the 120-kVp protocol in this study. The difference of the CT number and the image noise between 100-kVp protocol with HIR and 120-kVp protocol were relatively small,^[29]^ therefore the optimal display preset technique could easily adjust that difference.^[30]^

We believe that the combined use of the 100-kVp scan with moderate-level HIR is better technique in radiation dose reduction than the 100-kVp protocol with high-level HIR. The reason was that high-level HIR technique yields the over-smoothed image appearance, the loss of the classic image appearance and may prevent the accurate diagnosis. In addition, our 100-kVp protocol with moderate-level HIR with optimized display preset is superior in radiation dose reduction while preserving image quality. Previous studies suggested that the 100-kVp protocol could reduce the radiation dose only about 40% to 50% while preserving image quality compared with the conventional 120-kVp protocol.^[17,31]^ However, our 100-kVp protocol with moderate-level HIR with optimized display preset can reduce the 76% radiation dose while preserving image quality compared with 120-kVp protocol, because of HIR and optimal window setting.

There were some limitations in our study. First, we focused only on a moderate-level HIR technique and the 100-kVp technique. Theoretically, it would be desirable to evaluate the 80-kVp technique and a high-level HIR technique. However, many studies have reported that the 80-kVp and high-level HIR techniques cannot preserve the classic image appearance and decrease the image quality.^[32,33]^ Therefore, we did not evaluate these protocols regarding the advantages for patients. Second, the radiation dose exposure associated with our protocol was higher compared with the previous studies about the prospective ECG-gating scan because of using the retrospective ECG-gating scan.^[34–37]^ Although, for the patients with arrhythmia or tachycardia, a retrospective ECG-gating scan is necessary to evaluate the coronary artery accurately. Therefore, we believe that the 100-kVp protocol with HIR with optimized display preset is clinically useful in radiation dose reduction for retrospective ECG-gating scan. Third, we only enrolled the patients performed with retrospective ECG-gating scan in this study. Therefore, we did not establish that our 100-kVp protocol with HIR with optimized display preset is also useful for patients scanned with prospective ECG-gating. Lastly, this technique needs the iterative reconstruction technique and more than 128 rows MDCT or dual source CT, and cannot be apply in any machines. However, these CT machine and iterative reconstruction algorism might be introduced widely in no distant future.

## 5 Conclusion

The 100-kVp protocol with HIR with an optimized display preset can reduce the 76% radiation dose while preserving the image quality compared with the conventional 120-kVp protocol on retrospective ECG-gated cardiac CT.
